# Effects of neoadjuvant FOLFIRINOX and gemcitabine-based chemotherapy on cancer cell survival and death in patients with pancreatic ductal adenocarcinoma

**DOI:** 10.18632/oncotarget.27399

**Published:** 2019-12-31

**Authors:** Li Xie, Leizhou Xia, Ulla Klaiber, Milena Sachsenmaier, Ulf Hinz, Frank Bergmann, Oliver Strobel, Markus W. Büchler, John P. Neoptolemos, Franco Fortunato, Thilo Hackert

**Affiliations:** ^1^ Department of General, Visceral and Transplantation Surgery, University Clinic, Heidelberg, Germany; ^2^ Section Surgical Research, University Clinic, Heidelberg, Germany; ^3^ Institute of Pathology, University Clinic, Heidelberg, Germany; ^*^ Co-senior authors

**Keywords:** autophagy, cell death, stroma, neoadjuvant therapy, prognosis

## Abstract

**Background:** The progression and response to systemic treatment of cancer is substantially dependent on the balance between cancer cell death (apoptosis and necroptosis) and cancer cell survival (autophagy). Although well characterized in experimental systems, the status of cancer cell survival and cell death in human pancreatic ductal adenocarcinoma (PDAC), especially in response to chemotherapy and different types of chemotherapy is poorly described.

**Results:** The median (95% confidence interval) survival was 31.6 (24.5–44.5) months after FOLFIRINOX versus 15.8 (2.0–20.5) months after gemcitabine-based therapy (*p* = 0.039). PDAC tissue autophagy was reduced compared to normal pancreata based on reduced BECLIN-1 expression and LC3-Lamp-2 colocalization, whilst necroptosis (RIP-1) was increased. Neoadjuvant therapy was associated with further reduced autophagy based on p62/SQSTM-1 accumulation, and increased necroptosis (RIP3 and pMLKL) and apoptosis (BAX, cleaved CASPASE-9 and CASPASE-3) markers, increased nuclear p65 (NF-κB) and extracellular HMGB1 expression, with greater CD8^+^ lymphocyte infiltration. Survival was associated with reduced autophagy and increased apoptosis. Necroptosis (RIP-3, pMLKL) and apoptosis (BAX and cleaved CASPASE-9) markers were higher after FOLFIRINOX than gemcitabine-based treatment.

**Patients and methods:** Cancer cell autophagy, apoptosis, and necroptosis marker expression was compared in pancreatic tissue samples from 51 subjects, comprising four groups: (1) surgical resection for PDAC after FOLFIRINOX (*n* = 11), or (2) after gemcitabine-based (*n* = 14) neoadjuvant therapy, (3) patients undergoing PDAC resection without prior chemotherapy (*n* = 13), and (4) normal pancreata from 13 organ donors. Marker expression was undertaken using semi-automated immunofluorescence-FACS-like analysis, defining PDAC cells by CK-7^+^ expression.

## INTRODUCTION

Pancreatic ductal adenocarcinoma (PDAC) has a rising incidence with poor survival and is set to become the second commonest cause of cancer death [1, 2]. Surgery with adjuvant chemotherapy can provide cure but this is only possible in the 10-15% with resectable disease [1–4]. Most patients have metastatic disease in whom palliative chemotherapy is the mainstay of treatment, which includes combinations with gemcitabine or the FOLFIRINOX comprising folinic acid, 5-fluorouracil (5-FU), irinotecan and oxaliplatin [5–7]. There is increasing interest in neoadjuvant chemotherapy with borderline and locally irresectable disease [8]. Efforts are also focused on identifying specific signatures for different types of chemotherapy [9–13].

Different mechanisms of cell death are involved in the pathogenesis of PDAC and responses and resistance mechanisms to cytotoxicity. The contribution of these mechanisms including autophagy, necroptosis and apoptosis are complex with evolving concepts but with relatively few clinical studies. Cytotoxicity will cause DNA damage and induce apoptosis through the mitochondrial intrinsic pathway involving the activation of BAX-like proteins leading to unregulated calcium entry into the mitochondria with cytochrome C release, formation of the cytochrome C/APAF-1/CASPASE-9 apoptosome complex, and activation of the effector caspase-3 [14]. Cytotoxicity will also lead to the activation of receptor interacting kinases (RIP)-1 and RIP-3 leading to necrosome assembly, which in turn recruits mixed lineage kinase domain-like protein (MLKL) and its phosphorylation (pMLKL), then leading to necroptosis and the release of damage associated molecular patterns (DAMPs) [15].

More complex is the role of autophagy in the pathogenesis of PDAC and clinical correlates. Recruitment of autophagy-related gene ATG-5 is crucial in the formation of the autophagosome. This involves binding of ATG-5 to ATG-12-ATG-16 to mediate the palmitoylation (with phosphatidyl-ethanolamine) of microtubule-associated protein light chain 3 (LC3)-I to membrane-bound LC3-II, on the autophagosome. The p62 protein, also called sequestosome 1 (SQSTM-1), is a cargo receptor that binds ubiquitin on cargo in the phagophore or pre-autophagosome and cooperates with BECLIN-1 to deliver cargo to autophagosomes by docking onto LC3-II. The lysosomal-associated membrane proteins (LAMP)-1 and LAMP-2 and the small GTPase RAB-7 are involved in fusing the lysosome to the autophagosome to form the autolyosome [16]. Deletion of ATG-5 in the pancreas has been shown to increase tumor initiation but decrease tumor progression indicating a tumor stage-dependent action of autophagy [17–19]. Fujii *et al*., reported that activation of autophagy in PDAC was asscoiated with reduced survival [20]. On the other hand, TCGA-databank analysis by Görgülü *et al*., showed a reduction in ATG-5 copy numbers between human PDAC and normal pancreata, and found reduced survival with lower ATG-5 expression based on immunohistochemistry [19]. It has been highlighted however, that autophagic flux is difficult to measure in human tumor samples [21]. In order to clarify the status of cell survival and cell death in human PDAC we undertook a comprehensive analysis of multiple markers for each type of cell death using immunofluorescence-FACS-like quantitation analysis to objectify expression.

## RESULTS

**Figure 1:**
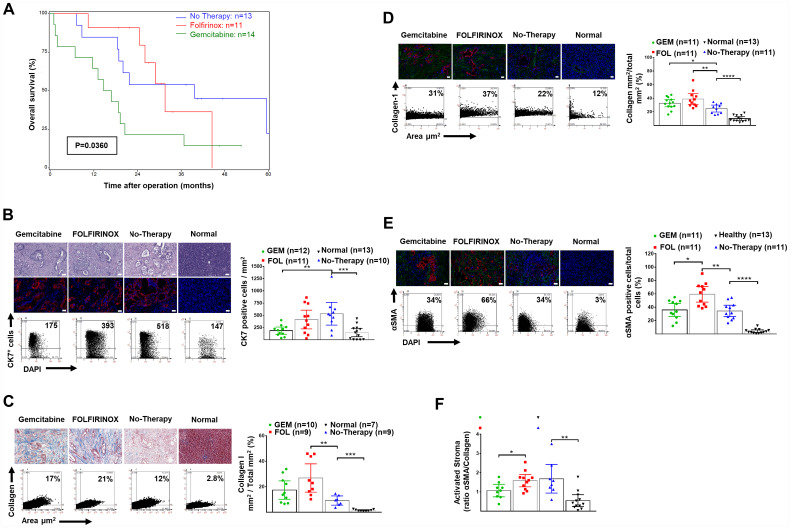
Overall survival and human pancreatic normal tissue and cancer stroma: H&E, CK7+ cells and expression of collagen-1 and αSMA. (**A**) Overall survival after resection and neoadjuvant FOLFIRINOX or gemcitabine-based chemotherapy in patients presenting with borderline or non-resectable pancreatic cancer. The median (95% confidence interval) survival was 31.6 (24.5–44.5) months after FOLFIRINOX (*n* = 11) versus 15.8 (2.0–20.5) months after gemcitabine-based therapy (*n* = 13) (*p* = 0.039). (**B**) Representative H&E stained tissues (top), and IF (bottom) for DAPI (blue) and CK-7^+^ tumor cells (red), as well as representative FACS-like co-expression scattergrams and quantitation of CK-7^+^ cells per area in mm^2^ are blotted as mean with 95% CI as shown in Table 2. (**C**) Representative trichrome stained tissues for collagen-1, as well as representative FACS-like co-expression scattergrams and quantitation of collagen-1 expression per area mm^2^ are blotted as mean with 95% CI as shown in Table 2. (**D**) Representative IF stained tissues for collagen-1 (green), DAPI (blue) and CK-7^+^ tumor cells (red), as well as representative FACS-like co-expression scattergrams and quantitation of collagen-1 expression per area mm^2^ are blotted as mean with 95% CI as shown in Table 2. (**E**) Representative IF stained tissues for αSMA (green), DAPI (blue) and CK-7^+^ tumor cells (red), as well as representative FACS-like co-expression scattergrams and quantitation of αSMA and are blotted as mean with 95% CI as shown in Table 2. (**F**) The mean (95% CI) activated stromal index (ratio of αSMA to collagen-1) by group. Human tissue scale bar = 20 μm, 20× objective. ^*^ *P* < 0.05, ^**^ *P* < 0.01, ^***^ *P* < 0.001, ^****^ *P* < 0.0001.

Clinicopathological variables are shown in Table 1. The grade of tumor differentiation was not determined after neoadjuvant because of the general inconsistency of interpretation. Overall median and 2-year survival was greater in patients given neoadjuvant therapy (Log-Rank χ^2^_df2_ = 6.6492, *p* = 0.0360) (Figure 1A).

**Table 1:** Clinical, histopathology and survival

Variable	No neoadjuvant therapy (control) (*n* = 13)	Three group comparison *P*-value	Gemcitabine-based neoadjuvant therapy (*n* = 14)	FOLFIRINOX neoadjuvant therapy (*n* = 11)	Gemcitabine vs FOLFIRINOX *P*-value
**Gender**		0.6528			1.0
**Male**	4 (30.8%)		7 (50.0%)	6 (54.5%)	
**Female**	9 (69.2%)		7 (50.0%)	5 (45.5%)	
**Median age (years)**	59.2	0.6747	59.6	58.4	0.3961
**range**	40.1–76.7		45.0 – 75.1	42.5–71.5	
**T-stage**		0.2975			0.2493
**pT1/ypT1**	0 (0.0%)		0 (0.0%)	1 (9.1%)	
**pT2/ypT2**	1 (7.7%)		0 (0.0%)	1 (9.1%)	
**pT3/ypT3**	12 (92.3%)		12 (85.7%)	9 (81.8%)	
**pT4/ypT4**	0 (0.0%)		2 (14.3%)	0 (0.0%)	
**Lymph node stage**		0.0782			0.6951
**pN0/ypN0**	2 (15.4%)		8 (57.1%)	5 (45.5%)	
**pN1/ypN1**	11 (84.6%)		6 (42.9%)	6 (54.5%)	
**Positive lymph node number**		0.1148			0.8545
**0**	2 (15.4%)		8 (57.1%)	5 (45.5%)	
**1-3**	5 (38.5%)		5 (35.7%)	4 (36.4%)	
≥**4**	6 (46.1%)		1 (7.1%)	2 (18.2%)	
**Metastasis stage**		0.2392			0.6043
**M0/ypM0**	13 (100%)		11 (78.6%)	10 (90.9%)	
**ypM1**	0 (0.0%)		3 (21.4%)	1 (9.1%)	
**Resection margin**		0.8076			0.8302
**R0**	4 (26.7%)		6 (42.9%)	4 (36.4%)	
**R1**	9 (73.3%)		7 (50.0%)	7 (63.6%)	
**Rx**	0 (0.0%)		1 (7.1%)	0 (0.0%)	
**UICC stage**		**0.0361**			0.5655
**UICC Ia**	0 (0.0%)		0 (0.0%)	1 (9.1%)	
**UICC Ib**	1 (7.7%)		0 (0.0%)	1 (9.1%)	
**UICC IIa**	1 (7.7%)		6 (42.9%)	3 (27.3%)	
**UICC IIb**	11 (84.6%)		4 (28.6%)	5 (45.5%)	
**UICC III**	0 (0.0%)		1 (7.1%)	0 (0.0%)	
**UICC IV**	0 (0.0%)		3 (21.4%)	1 (9.1%)	
**Survival**					
**Median overall**	31.6	Log rank	15.8	39.7	Log rank
**(months)**		x^2^_2df_ = 6.64			x^2^_1df_ = 64.26
**95% CI**	24.5–44.5	***p* = 0.0360 **	2.0–20.5	18.5–59.5	***p* = 0.0390 **
**2 Year (%)**	53.8		21.4	79.5	
**95% CI**	24.9–76.0		5.2–44.8	39.3–94.5	

We determined the content of CK7^+^ tumor cells within the PDAC and healthy pancreatic tissue using immunofluorescence as previously reported [22–24]. Tumors from patients following neoadjuvant therapy had significantly fewer CK7^+^ tumor cells compared to tumors from patients without prior chemotherapy (Table 2 and Figure 1B).

**Table 2:** Markers of cell death in normal and pancreatic cancer tissue and following neoadjuvant therapy

Markers	Normal Pancreas (*n* = 13)	^1^ *P* value Normal vs Control	No neoadjuvant therapy (control) (*n* = 13)	Control vs Gemcitabine vs FOLFIRINOX *^2^P*	Gemcitabine- based neoadjuvant therapy (*n* = 14)	Gemcitabine vs FOLFIRINOX *^1^P*	FOLFIRINOX neoadjuvant therapy (*n* = 11)
	**Median (95% CI)**		**Median (95% CI)**		**Median (95% CI)**		**Median (95% CI)**
**CK7^+^ cells / per mm^2^**	147.0 (8.00–244.0)	**0.0006**	518.5 (197.0–673.0)	**0.0158**	195.0 (53.00–298.0)	NS	393.0 (99.00–773.0)
**Collagen mm^2^ / Area mm^2^ (%)**	7.7 (6.87–14.9)	**<0.0001**	25.9	**0.0117**	32.8	NS	36.52
			16.49-32.96		27.59–41.56		8.4–51.2
**αSMA^+^ cells/ total cells %**	2.9	**<0.0001**	31.3	**0.0067**	33.4	**0.0128**	61.8
	1.62–6.82		20.95–52.39				40.73–75.27
**Ratio αSMA/ Collagen**	0.400 (0.210–0.850)	**0.0015**	1.485 (0.600–3.500)	NS	1.070 (0.730–1.500)	**0.0361**	1.610 (1.040–2.120)
**Autophagy Markers**							
**p62/SQSTM1 in CK7^+^ cells %**	34.10 (4.70–93.90)	NS	53.50 (6.600–77.60)	**0.0004**	86.05 (62.50–97.10)	NS	94.60 (93.40–98.00)
**BECLIN1^+^ cells/ total cells %**	33.18 (19.16–52.14)	**0.0056**	4.032 (0.28–17.67)	NS	2.91 (0.34–9.98)	NS	5.59 (3.15–15.25)
**ATG5 in CK7^+^ cells %**	85.79 (72.72–90.85)		84.04 (55.42–90.11)	NS	88.34 (75.11–96.05)	NS	69.41 (51.40–84.05)
**ATG7 in CK7^+^ cells %**	56 (36.72–76.20)	NS	80 (68.83–93.16)	NS	66 (45.25–86.81)	NS	61 (38.31–84.73)
**LC3 and LAMP2^+^ cells %**	43.82 (14.46–62.21)	**0.0140**	12.94 (1.882–41.47)	NS	16.03 (0.820–19.73)	NS	12.60 (4.793–17.78)
**Necroptosis Markers**							
**RIP1 in CK7^+^ cells %**	11.07 (4.240–22.39)	**0.0048**	71.49 (15.91–78.86)	NS	69.31 (44.88–87.36)	NS	80.20 (44.85–98.85)
**RIP3 in CK7^+^ cells %**	7.080 (3.400–64.17)	NS	39.48 (6.070–56.72)	**0.0002**	70.87 (36.57–90.09)	**0.0243**	94.87 (77.00–98.92)
**pMLKL in CK7^+^ cells %**	3.900 (0.080–14.30)	NS	0.4000 (0.150–4.180)	**< 0.0001**	4.495 (2.70–16.15)	**0.0022**	19.08 (8.60–39.49)
**Apoptosis Markers**							
**BAX in CK7^+^ cells %**	14.62 (0.610–40.22)	NS	14.53 (3.000–45.15)	**0.0004**	44.16 (27.24–73.04)	**0.0299**	75.27 (45.05–93.73)
**Cleaved Caspase-9 in CK7^+^ cells %**	40.46 (2.810–61.67)	NS	30.06(6.98–48.42)	**0.0008**	61.09 (41.98–78.55)	**0.0148**	83.76 (72.77–90.86)
**Caspase-3 in CK7^+^ cells %**	6.100 (0.920–20.01)	NS	4.145 (0.540–21.29)	**0.0007**	27.97 (11.97–55.40)	NS	52.87
							(30.50–66.59)
**Inflammatory cell infiltrate Markers**							
**Extracellular HMGB1 per area %**	0.720 (0.0671–1.506)	NS	1.361 (0.00–2.989)	**0.0102**	3.027 (1.004–19.36)	NS	3.424 (1.558–23.14)
**Macro-Φ cells %**	0.205 (0.110–0.590)	**0.0052**	0.870 (0.410–5.270)	NS	1.430 (0.910–2.060)	NS	1.340 (0.530–5.270)
**CD8^+^ cells %**	0.39 (0.25–0.74)	NS	0.95 (0.43–1.47)	**0.0016**	2.05 (1.11–4.65)	NS	4.06 (2.61–6.48)
**MPO Expression %**	0.175 (0.090–0.920)	**0.0205**	0.665 (0.230–1.94)	NS	0.65 (0.37–2.810)	NS	2.00 (0.680–4.540)
**NF-κB p65 in CK7^+^ cells %**	19.01 (1.85–38.06)	**NS**	33.37 (7.93–61.66)	**0.0008**	80.20 (70.24–92.29)	NS	83.81 (53.91–89.33)

^1^2 tailed Mann-Whitney *U* test; ^2^Kruskal-Wallis test.

Tumors had significantly more stromal collagen-I and activated αSMA^+^ cells per unit area compared to normal tissues with a corresponding higher collagen-I to αSMA^+^ cells ratio, or activated stromal index. There was even greater collagen-I deposition and αSMA^+^ cell activation after chemotherapy, which was especially marked after FOLFIROINOX therapy compared to neoadjuvant gemcitabine (Table 2, Figure 1C–1F). In patients who had neoadjuvant therapy the Activated Stromal Index (ratio αSMA/collagen), using the median cut off value = 1.3 for stratification was not associated with survival (Log-Rank χ^2^_df1_ = 0.0105, *p* = 0.9184).

**Figure 2:**
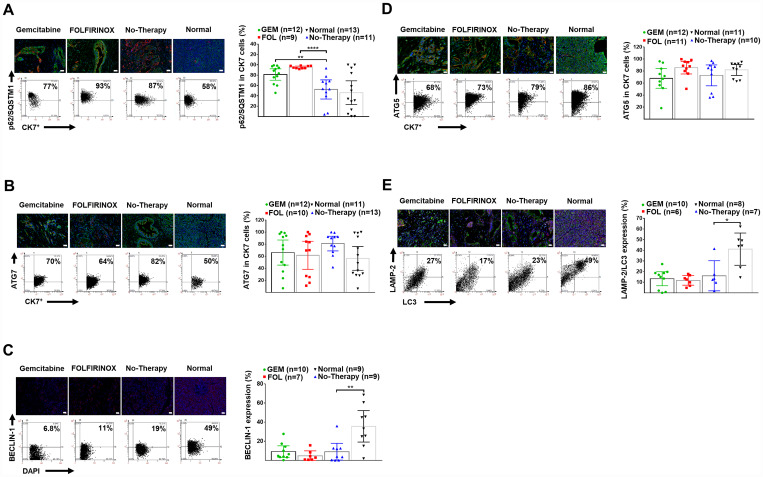
Human pancreatic normal and cancer tissues: expression of autophagy markers. (**A**) Representative IF-stained tissues for DAPI (blue), CK-7^+^ tumor cells (red) and p62/SQSTM-1 (green), as well as representative FACS-like co-expression scattergrams and quantitation of p62/SQSTM-1 in CK-7^+^ tumor cells are blotted as mean with 95% CI as shown in Table 2. (**B**) Representative IF-stained tissues for ATG-7 (green), DAPI (blue), and CK-7^+^ tumor cells (red), as well as representative FACS-like co-expression scattergrams and quantitation of ATG-7 in CK-7^+^ tumor cells are blotted as mean with 95% CI as shown in Table 2. (**C**) Representative IF-stained tissues with or for BECLIN-1 (red) and DAPI (blue), as well as representative FACS-like co-expression scattergrams and quantitation of BECLIN-1 in DAPI cells are blotted as mean with 95% CI as shown in Table 2. (**D**) Representative IF-stained tissues for ATG-5 (green), DAPI (blue), and CK-7^+^ tumor cells (red), as well as representative FACS-like co-expression scattergrams and quantitation of ATG-5 in CK-7^+^ tumor cells are blotted as mean with 95% CI as shown in Table 2. (**E**) Representative IF-stained tissues for LAMP-2 (red), LC-3 (green) and DAPI (blue), as well as representative FACS-like co-expression scattergrams and quantitation of LC-3 with LAMP-2 are blotted as mean with 95% CI as shown in Table 2. Human tissue scale bar = 20 μm, 20× objective. ^*^ *P* <0.05, ^**^ *P* < 0.01, ^***^ *P* < 0.001, ^****^ *P* < 0.0001.

There was reduced autophagy in human PDAC tissue compared to normal pancreata as shown by decreased expression of BECLIN-1, along with decreased colocalization of LC-3 with LAMP-2. Chemotherapy caused further inhibition as shown by an accumulation of p62/SQSTM-1 in CK-7^+^ tumor cells (Table 2, Figure 2A–2E). In patients who had neoadjuvant therapy there was increased survival associated with reduced autophagy based on the expression of BECLIN1 (median cut off level =10, Log-Rank χ^2^_df1_ = 5.2965, *p* = 0.0214), and p62/SQSTM-1 (median cut off level = 80, Log-Rank χ^2^_df1_ = 4.7197, *p* = 0.0298), and colocalization of LC-3 with LAMP-2 (median cut off level = 21, Log-Rank χ^2^_df1_ = 3.7672, *p* = 0.0523).

**Figure 3:**
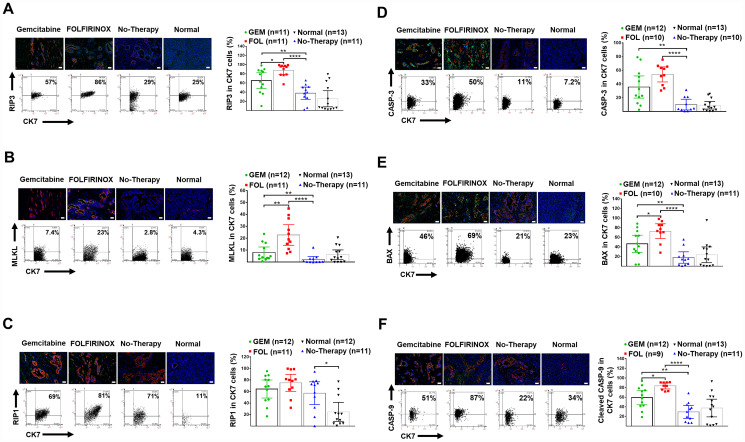
Human pancreatic normal and cancer tissues: expression of necroptosis and apoptosis markers. (**A**) Representative IF-stained tissues for DAPI (blue), CK-7^+^ tumor cells (red), and RIP-3 (green), as well as representative FACS-like co-expression scattergrams and quantitation of RIP-3 in CK-7^+^ tumor cells are blotted as mean with 95% CI as shown in Table 2. (**B**) Representative IF-stained tissues for DAPI (blue), CK-7^+^ tumor cells (red), and pMLKL (green), as well as representative FACS-like co-expression scattergrams and quantitation of pMLKL in CK-7^+^ tumor cells are blotted as mean with 95% CI as shown in Table 2. (**C**) Representative IF-stained tissues for DAPI (blue), CK-7^+^ tumor cells (red), and RIP-1 (green), as well as representative FACS-like co-expression scattergrams and quantitation of RIP-1 in CK-7^+^ tumor cells are blotted as mean with 95% CI as shown in Table 2. (**D**) Representative IF-stained tissues for CASPASE-3 (green), DAPI (blue) and CK-7^+^ tumor cells (red), as well as representative FACS-like co-expression scattergrams and quantitation of CASPASE-3 in CK7^+^ tumor cells are blotted as mean with 95% CI as shown in Table 2. (**E**) Representative images of pancreatic tumor stained for BAX (green), DAPI (blue) and CK7^+^ tumor cells (red) as well as representative FACS-like co-expression scattergrams and quantitation of BAX in CK7^+^ tumor cells are blotted as mean with 95% CI as shown in Table 2. (**F**) Representative images of pancreatic tumor tissue stained for cleaved CASPASE-9 (green), DAPI (blue) and CK7^+^ tumor cells (red), as well as representative FACS-like co-expression scattergrams and quantitation of CASPASE-9 in CK7^+^ tumor cells are blotted as mean with 95% CI as shown in Table 2. Human tissue scale bar = 20 μm, 20× objective. ^*^ *P* < 0.05, ^**^ *P* < 0.01, ^***^ *P* < 0.001, ^****^ *P* < 0.0001.

Necroptosis was shown to be increased in human PDAC tissue compared to normal pancreata as shown by increased expression of RIP-1 in CK-7^+^ cells. Necroptosis was further enhanced with chemotherapy as shown by increased expression of RIP-3 and pMLKL (Table 2, Figure 3A–3C).

Apoptosis was not increased in human PDAC tissue without a therapy compared to normal pancreata. Apoptosis was increased following chemotherapy and was especially marked following chemotherapy with FOLFIRINOX compared to gemcitabine (Table 2, Figure 3D–3F). In patients who had neoadjuvant therapy there was increased survival associated with increased apoptosis based on the expression of BAX (median cut off level = 45.2, Log-Rank χ^2^_df1_ = 16.3416, *p* < 0.0001).

**Figure 4:**
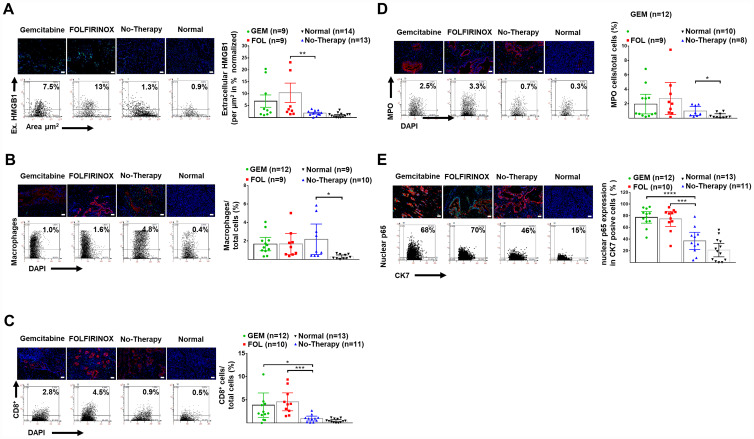
Enhanced Inflammation after neoadjuvant therapy. (**A**) Representative images of human pancreatic tissue stained for DAPI (blue) and extracellular HMGB1 (green), as well as representative FACS-like scattergrams and quantitation values of normalized extracellular HMGB1 expression per tissue area are blotted as mean with 95% CI as shown in Table 2. (**B**) Representative images of human pancreatic tissue stained for DAPI (blue), macrophages (green) and CK7^+^ tumor cells (red), as well as representative FACS-like scattergrams and quantitation values of macrophages are blotted as mean with 95% CI as shown in Table 2. (**C**) Representative images of human pancreatic tissue stained for DAPI (blue), CD8^+^ TILs (green) and CK7^+^ tumor cells (red) as well as representative FACS-like scattergrams and quantitation of CD8^+^ TILs are blotted as mean with 95% CI as shown in Table 2. (**D**) Representative images of human pancreatic tissue stained for MPO (green), (green), DAPI (blue) and CK7^+^ tumor cells (red), as well as representative FACS-like scattergrams and quantitation values of MPO are blotted as mean with 95% CI and shown in Table 2. (**E**) Representative images of human pancreatic tissue stained for nuclear p65 (part of NFкB) (green), DAPI (blue) and CK7^+^ tumor cells (red) as well as representative FACS-like scattergrams and quantitation values of nuclear p65 are blotted as mean with 95% CI and shown in Table 2. (Scale bar = 20 μm, 20× objective). ^*^ *P*< 0.05, ^**^ *P* < 0.01, ^***^ *P* < 0.001, ^****^ *P* < 0.0001.

There was an enhanced inflammatory response in pancreatic tumors and after chemotherapy with a significant increase in extracellular HMGB-1, and stromal infiltration by CD-8^+^ T-lymphocytes in human PDAC tissue. Tumor infiltration by macrophages and neutrophils was significantly increased in tumor tissue compared to controls. Expression of the nuclear transcription factor NFкB p65 in in CK-7^+^ tumor cells was also greatly increased following chemotherapy (Table 2, Figure 4A–4E).

## DISCUSSION

In this study we found that pancreatic cancer epithelial cells had reduced autophagy compared to normal epithelial cells and was further reduced by chemotherapy. Decreased autophagy induced by chemotherapy was associated with improved survival. FOLFIRINOX also induced greater necroptosis and apoptosis than gemcitabine based-therapy, which may also contribute to longer survival.

Various animal models show that inhibition of autophagy increases DNA damage, suppresses pancreatic cancer cell growth, and involves both tumor cell-intrinsic and host effects [18]. Experimentally the addition of chloroquine to single chemotherapy agents does not always increase treatment efficacy and chloroquine inhibition of autophagy in cancer associated fibroblasts may increase cancer cell resistance to gemcitabine [25]. On the other hand, Bryant *et al.* have shown that suppression of KRAS or the downstream effectors ERK-MAPK increased autophagic flux, with decreased glycolytic and mitochondrial functions, and that inhibition of both ERK-MAPK and autophagy may be an effective treatment for PDAC [26].

Studies of cell survival and cell death in patients with PDAC with clinical outcomes are relatively few. In 2008 Fujii S, *et al.* found that strong LC3 expression in the peripheral area of resected pancreatic cancer tissue was associated with reduced survival compared to weak or negative expression [20]. Koh *et al*. also reported that BECLIN-1 overexpression was associated with poor prognosis [27]. Boone *et al*. in a phase I/II trial of neoadjuvant hydroxyl-chloroquine with gemcitabine in 35 patients with PDAC, found that patients who had more than a 51% increase in LC3-II in circulating peripheral blood mononuclear cells had an improvement in overall survival but no assessment was made of in the resected tumor specimens [28]. Görgülü *et al*., also reported that lower levels of ATG5 were associated with both tumor metastasis and shorter survival time [19].

This is the only study in humans to compare a range of autophagy proteins in normal pancreata, untreated PDAC tissues and in PDAC tissues after two types of commonly used chemotherapy. The results demonstrated a reduction in autophagy by neoadjuvant chemotherapy compared to untreated PDAC, and longer survival associated with reduced autophagy. Unlike the study of Görgülü *et al*., we found no association between survival and expression of ATG-5 and ATG-7 in CK7^+^ cells, whilst the other autophagy markers indicated an opposite finding.

There may be a number of reasons for his discrepancy including different methodologies and different patient cohorts. Our findings support the conclusion from experimental studies that reduced autophagy, whether intrinsic, genetically, or pharmacologically induced, is associated with improved survival [18, 26, 28–31].

We found no significant association between the activated stromal index (ratio αSMA/collagen) and survival, and perhaps more surprisingly no difference between chemo-naive PDAC and post induction chemotherapy [32]. We found that neoadjuvant therapy was associated with further increased necroptosis and apoptosis marker expression as well as increased nuclear p65 (NF-κB) and extracellular HMGB1 expression. Moreover necroptosis and apoptosis markers were higher after FOLFIRINOX than gemcitabine-based treatment reflecting the better respective response and survival rates [4–8]. These findings are largely original in human PDAC tissue. It was interesting to see that necroptosis was particularly induced by neoadjuvant FOLFIRINOX as this mechanism may overcome intrinsic or gemcitabine induced resistance to apoptosis. It should be noted that excess necroptosis with DAMPs, may result pro-tumorigenic inflammation and immunosuppression [15]. The necrosome may represent an important therapeutic target for PDAC as necroptosis, may be pharmacologically induced by the aurora kinase inhibitor CCT137690 [33]. Although PDAC is highly resistant to apoptosis, this study showed a considerable increase in apoptosis with chemotherapy which was greatest with FOLFIRINOX [14, 34]. Longer survival was also associated with increased apoptosis. There were significantly more tumor infiltrating CD8^+^ cytotoxic T lymphocytes after neoadjuvant therapy which favors improved survival [35, 36].

Taken together these results indicate a favorable response to neoadjuvant chemotherapy and in particular FOLFIRINOX, including reduced autophagy, and increased necroptosis, apoptosis and CD8^+^ tumor infiltration. We found that decreased PDAC tissue autophagy and increased apoptosis, were associated with longer survival after neoadjuvant chemotherapy. These findings support targeting autophagy as a therapeutic strategy against pancreatic cancer. In addition, clinical studies testing anti-autophagy therapies should employ a range of cancer tissue autophagy markers.

## MATERIALS AND METHODS

### Patients and study design

We identified four groups from the pancreas clinical database and biobank at the Department of Surgery, Heidelberg: (1) patients with initially locally advanced unresectable PDAC (without metastases) who then had surgical resection after FOLFIRINOX (*n* = 11) neoadjuvant therapy, (2) similar patients with initially unresectable PDAC who then had surgical resection for PDAC after gemcitabine-based (*n* = 14) neoadjuvant therapy, (3) patients who had resectable PDAC at presentation and had up-front surgical resection without prior chemotherapy (*n* = 13), and (4) normal pancreata from 13 organ donors. We only included patients with PDAC who had good quality tumor tissue and who had completed a full neoadjuvant course of either FOLFIRONOX or gemcitabine-based chemotherapy. Clinic-pathological variables including survival time were only analyzed at the completion of the study and were not assessed in the selection procedure.

The overall median survival was compared in the two groups with unresectable PDAC and related to the expression of cell death markers. The survival of patients with resectable PDAC is also provided but cannot be directly compared survival of with the patients with initially non-resectable tumors. This study was approved by the Ethics Committee of Heidelberg University (Ethics Committee Approval No. 301/2001, renewed 2012).

### Histopathology

Fresh tissue samples were immediately fixed in 4% buffered formalin for approximately 24 h, then immersed in 70% ethanol for 2 days and embedded by paraffin into blocks. The formalin-fixed paraffin-embedded (FFPE) blocks were stored at 4°C and then cut into 4 μm thick sections using a Leica microtome and stained with Hematoxylin (H) (VWR International Darmstadt, Germany) and Eosin (E) (Carl Roth, Karlsruhe, Germany).

Tumor specimens and H and E slides were checked for diagnosis and tumor cellularity in the Department of Pathology, and anonymised. Staging was undertaken using the UICC 7th Edition [37]. Masson-Goldner’s trichrome staining kit (Carl Roth, Karlsruhe, Germany) was used to stain for collagen-1 and α-smooth-muscle actin (αSMA) in order to determine activated stroma index [32].

Antibodies were chosen for immunofluorescence (IF) to permit quantification of PDAC epithelial cells using cytokeratin (CK)-7 expression [24]. The autophagy markers used included BECLIN-1, ATG-5 and ATG-7. We also used p62/SQSTM-1 as a marker of autophagic flux as levels accumulate when autophagy is inhibited, and are reduced when autophagy is activated [38]. There are no antibodies that distinguish LC3-I from LC3-II, and so colocalization of LC-3 with LAMP-2 was used to indicate autophagy activation with the formation of autolysosomes [16]. The necroptosis markers used were RIP1 and RIP3 and pMLKL. The apoptosis markers used were BAX, CASPASE-3 and cleaved CASPASE-9. As well as using antibodies for identifying stromal infiltration by macrophages, and CD-8^+^ lymphocytes, we also used myeloperoxidase (MPO) staining to determine inflammatory cell infiltration. We included detection of high-mobility group box 1 (HMGB-1), which is associated with autophagy and is a DAMP molecule [39]. We included a marker for NF-κB p65, which activates the receptor for advanced glycation end products (RAGE) under stress, and induces autophagy [40].

### Immunofluorescence

FFPE 4 μm thin sections were deparaffinised and rehydrated with Roticlear and Ethanol series (Carl Roth, Karlsruhe, Germany). Antigen retrieval was achieved by placing the sections in sodium citrate buffer in a water bath at 98°C for 10 min. After cooling at 20°C for 30 min, the sections were rinsed in Tris-buffered saline (TBS) for 5 min and permeabilized with 0.1% saponin in TBS at 20°C for 20 min. The sections were washed with 0.05% Tween-20 in TBS for 2min and then circumscribed with a Cytomation Dako Pen (Dako, Hamburg, Germany). Target specific primary antibodies were incubated in antibody diluent solution (Dako, Hamburg, Germany) for 30 min at 37°C in a dark humidified chamber, followed by several steps of washing. Sections were then incubated with secondary anti-mouse Cy5 and/or anti-rabbit Cy3 and/or goat-Cy2 labelled antibodies for 30 min at 20°C, followed by several steps of washing and incubation with nuclei counterstained using 4ʹ-6-diamidino-2-phenylindole (DAPI) for 20 min. The sections were mounted in Fluoromount-G Reagent (Southern Biotech, Birmingham, AL, USA). We used the TissueGnostics Fluorescence Imaging System (TissueGnostics, Vienna, Austria), with a fluorescence microscope unit (Observer. Z1, Zeiss), and an X-Cite^®^ Series 120PC dynamic fluorescence illuminator (X-Cite^®^, Ontarioo, Canada). The TissueFAXS Imaging Software module automatically captured images by a 20× objective using different channels to detect the target proteins, with controlling filters, exposure, camera (PCO, Kehlheim, Germany), and motor stage (Märzhäuser, Wetzlar, Germany). StrataQuest Analysis Software (TissueGnostics, Vienna, Austria) was used to produce FACS-like analysis quantitation from IF images, comparing Cy3 (green) and/or Cy5 (red) intensity with DAPI intensity. Cy5- and Cy3-positive cells were gated in the scattergrams according to negative controls without primary antibody. We also used this system to acquire the images of H&E and trichrome staining and has been described previously [16, 41–43].

### Antibodies and reagents

For immunofluorescence we used the following antibodies. α-SMA (sc-32251), CK7 (sc-23876), p62/SQSTM1 (sc-25575), BECLIN1 (sc-48341), ATG7 (sc-8668), BAX (sc-526), LAMP-2 (sc-5571), macrophage marker (sc-66204), and Erk1/2 (sc-154) were all purchased from Santa Cruz Biotechnology (Heidelberg, Germany). Anti-Collagen-1 (ab-34710) was purchased from Abgent (San Diego, USA). Collagen-I (ab-34710), ATG5 (AP1812a), LC3 (AM1800a), RIP3 (ab-72106), p-MLKL (ab-187091), CASPASE-3 (ab-2171), HMGB1 (ab12029), CD8 (ab-4055), and MPO (ab-9535) were purchased from Abgent (San Diego, USA). RIP1 (NPB1-77077), cleaved CASPASE-9 (NB100-56118), and NF-κB p65 (Ser276) (SAB-11011) were purchased from Novus Biologicals (Cambridge, UK). Secondary anti-rabbit Cy3- or Cy5- conjugated and anti-mouse Cy3- or Cy5- conjugated antibodies were purchased from Medac GmbH (Wedel, Germany). All other chemicals, unless stated otherwise, were obtained from Merck, Darmstadt, Germany.

### Statistical analysis

We estimated overall survival using the Kaplan-Meier method, determined from the date of pancreatic surgery to the censor point or death from any cause. Median survival times and 3-year survival rates with 95% confidence intervals (CI) are presented, using the log-rank test for comparison. Continuous variables are presented as the median with 95% CI, with comparison by the Kruskal-Wallis test for multiple groups and the Mann-Whitney *U* test for two group comparisons. Significance was set at a two-sided *p* ≤ 0.05. We used GraphPad Prism 6 and SAS software, Release 9.4, SAS Institute, Inc, Cary, NC, USA. The graphs were presented as the mean with 95% CI. All results were reported as median 95% CI, as indicated with the significance score (^*^<0.05; ^**^<0.01; ^***^<0.001, ^****^<0.0001) in the figure legends.
